# Prevalence, multiterritorial vascular distribution, and burden of subclinical atherosclerosis in psoriasis: The EDSAP study

**DOI:** 10.1016/j.jdin.2025.11.026

**Published:** 2025-12-16

**Authors:** Carlota Abbad-Jaime de Aragon, Emilio Berna-Rico, Alba Lecumberri, Javier Perez-Bootello, María Davó-Mogica, Fernando Neria-Serrano, Diana Monge, María Asunción Ballester-Martínez, Cristina Pindado-Ortega, María Castellanos-González, Mar Llamas-Velasco, Samantha Wasniewski, María G. Barderas, Pedro Jaén, Joel M. Gelfand, Nehal N. Mehta, Jorge Solís, Leticia Fernández-Friera, Álvaro González-Cantero

**Affiliations:** a Department of Dermatology, Hospital Universitario Ramón y Cajal, IRYCIS, Madrid, Spain; b Faculty of Medicine, Universidad Francisco de Vitoria, Madrid, Spain; c Department of Dermatology, Hospital del Sureste, Madrid, Spain; d Department of Dermatology, Hospital Universitario La Princesa, Madrid, Spain; e Atria Clinic, Madrid, Spain; f HM CIEC Madrid (Centro Integral de Enfermedades Cardiovasculares), Hospital Universitario HM Montepríncipe, HM Hospitales, Madrid, Spain; g Facultad HM de Ciencias de la Salud de la Universidad Camilo José Cela, Madrid, Spain; h Instituto de Investigación Sanitaria HM Hospitales, Madrid, Spain; i Department of Vascular Physiopathology, Hospital Nacional de Parapléjicos, IDISCAM, Toledo, Spain; j Department of Vascular Physiopathology, Hospital Nacional de Parapléjicos, SESCAM, Toledo, Spain; k Department of Biostatistics, Epidemiology and Informatics, Perelman School of Medicine, University of Pennsylvania, Philadelphia, Pennsylvania; l Department of Dermatology, Perelman School of Medicine, University of Pennsylvania, Philadelphia, Pennsylvania; m Department of Cardiology, George Washington Medical Center, Washington, DC, USA; n Department of Cardiology, Hospital Universitario 12 de Octubre, Madrid, Spain

**Keywords:** atherosclerosis, cardiovascular disease, inflammation, psoriasis, epidemiology

## Abstract

**Background:**

Psoriasis increases atherosclerosis risk due to inflammation. To date, subclinical atherosclerosis (SA) in psoriasis has only been studied in individual vascular territories by imaging.

**Objective:**

To conduct a comprehensive evaluation of multiterritorial SA prevalence in psoriasis by imaging and establish its relationships with cardiovascular (CV) risk scores.

**Methods:**

A total of 120 patients with moderate-to-severe psoriasis without CV disease from the Early Detection of Subclinical Atherosclerosis in Psoriasis (EDSAP) cohort underwent vascular ultrasound of carotid/femoral arteries and noncontrast/contrast-coronary computed tomography angiography. SA was defined by the presence of any plaque or a coronary artery calcium score ≥1.

**Results:**

The median age was 48.04 (8.25) years, 73% were male, and 77% of participants had SA. Femoral arteries were most affected (57.1%), followed by the coronaries (51.3%) and carotid arteries (49.6%). Femoral plaques exhibited the strongest associations with coronary parameters. CV risk scores underestimated SA, as at least 60% low-risk and 90% moderate-risk patients had SA.

**Limitations:**

The main limitation is the small sample size.

**Conclusions:**

This study provides the first multiterritorial assessment of SA in psoriasis, revealing a high prevalence of early disease. Femoral arteries were most affected, correlating with coronary atherosclerosis. The high SA detection within low-/intermediate-risk individuals suggests recalibrating CV scores for establishing effective preventive measures.

## Graphical abstract

**Figure:**
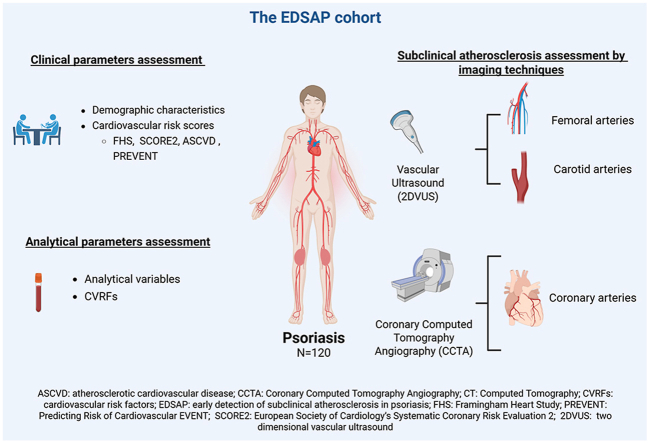

**Capsule Summary**

• This research presents evidence to support the reclassification of cardiovascular risk stratification in psoriasis, suggesting techniques to improve the prediction of coronary atherosclerosis risk, particularly in individuals deemed low risk by conventional methodologies.
• The findings could help facilitate informed clinical decision-making and earlier therapeutic interventions, contributing to improved patient outcomes.

## Introduction

Psoriasis is associated with a 4-year decrease in life expectancy as a result of an accelerated subclinical atherosclerosis (SA), which can lead to increases in cardiovascular (CV) events at younger ages than the general population.^1^^,^^2^

Despite the recognized systemic inflammatory role in psoriasis, CV disease (CVD) association,^3^^,^^4^ clinical risk stratification remains limited. This is due to infrequent early atherosclerosis assessment and traditional scores underestimating CV risk in this inflamed population.^5^^,^^6^ Cardiology guidelines now include psoriasis as an independent CVD risk factor (CVRF) and recommend statin therapy for patients with intermediate-risk psoriasis based on CV scores.^7^^,^^8^

The use of vascular imaging techniques could help to bridge this gap. Atherosclerosis, the main cause of mortality worldwide, develops decades before fatal events occur.^9^ Plaque detection through noninvasive imaging techniques has become the cornerstone for identifying atherosclerosis in asymptomatic individuals (SA), allowing for the implementation of primary prevention strategies. Considering the systemic nature of atherosclerosis, a multiterritorial approach may be crucial for a comprehensive disease evaluation. The Progression of Early Subclinical Atherosclerosis study exemplifies this approach by using imaging techniques to assess SA across multiple vascular territories, revealing a high SA prevalence in asymptomatic middle-aged adults from the general population. So far, atherosclerosis detection in psoriasis through imaging has been limited to individual vascular territories (either peripheral or coronary alone),^6^^,^^10^, ^11^, ^12^ lacking a multiterritorial approach, which would allow to better understand SA distribution within a highly inflamed population, including peripheral and coronary arteries, which is associated with a poorer prognosis in psoriasis, and may allow for a more accurate stratification of CV risk.

The aim of this study is to assess SA prevalence, distribution, and burden in psoriasis using vascular ultrasound (VUS) of peripheral arteries and coronary computed tomography angiography (CCTA) in the Early Detection and Progression of Subclinical Atherosclerosis in Psoriasis (ESDAP) cohort.^13^ We also aim to compare the effectiveness of established CV risk scores and these imaging modalities for early SA detection in the psoriasis population, to identify the best CV risk stratification methods.

## Methods

### Study population

This study included 120 patients with moderate-to-severe psoriasis from the EDSAP cohort, with a protocol previously reported and approved (HIP/CI-BIOB-058-01).^13^ The study followed Strengthening the Reporting of Observational Studies in Epidemiology guidelines.^14^ Patients did not have prior clinical history of CVD, met the inclusion/exclusion criteria, and were examined for the assessment of CVRFs, clinical risk scores (atherosclerotic cardiovascular disease [ASCVD], systematic coronary risk evaluation-2 [SCORE2], the 10-year risk of coronary heart disease from the Framingham Heart Study [FHS], and predicting 10-year risk of cardiovascular disease events [PREVENT]), and blood sampling (Supplementary Methods, available via Mendeley at https://data.mendeley.com/datasets/wzptvv27vx/1).

### SA assessment and definition

All patients underwent screening for SA. The coronary territory was assessed by noncontrast computed tomography (CT) and CCTA imaging, performed with a 320-detector CT scanner (Aquilion ONE ViSION) at the Hospital Universitario HM Sanchinarro (Madrid, Spain). Coronary artery calcium score (CACS) was calculated with the Agatston method. Acquisition of 2DVUS images in the femoral and carotid arteries was performed with a Philips iU22 ultrasound system (Philips Healthcare) at Hospital Universitario 12 de Octubre (Madrid, Spain). Detailed information on image acquisition procedures is provided in the Supplementary Methods section. SA was defined as the presence of atherosclerotic plaques in the carotid, femoral, or coronary arteries, or as CACS ≥ 1. SA extent was categorized by affected vascular territories (carotids, femorals, and coronaries). Patients were grouped as disease-free (0 sites), focal (1 site), or multivessel (2-5 sites). For the SA burden analysis, patients were divided into low (0-1 sites) and high (2-5 sites).

## Statistical analysis plan

Data were presented as mean ± SD (normal distribution) or median and IQR (skewed). Linear regression analyzed continuous variables’ association with SA, whereas logistic regression assessed categorical variables and peripheral plaque presence with coronary parameters. Associations between SA in the femoral and carotid territories were examined using logistic regression models adjusted for age and sex. Diagnostic performance, including positive predictive value and negative predictive value (NPV, respectively), was evaluated if carotid and femoral plaques indicated a higher likelihood of obstructive coronary plaque. Analysis of variance and post hoc tests compared patient groups with free/focal/multiple vascular territory involvement. Free/focal groups were combined due to similarities, creating low (free/focal) and high (multiple territories) SA burden groups, facilitating clearer comparison of clinical and demographic characteristics between groups (Table I).

**Table I:** Results of the post hoc analysis (Bonferroni test) for the comparison of basal characteristics between the groups of patients with different territories affected (focal, free, and multiple)

Patient data	Free (N = 27)	Focal (N = 27)	Multiple (N = 65)	*P* value	Free vs focal	Free vs multiple	Focal vs multiple
Demographics and clinical characteristics							
Age, y	41.48 (6.36)	44.40 (6.69)	52.43 (6.88)	**<.001**	0.339	**<0.001**	**<0.001**
Male, n (%)	21 (78%)	18 (66.6%)	48 (74%)	.68	0.362	0.692	0.486
BMI, kg/m^2^	25.76 (23.60-28.27)	26.72 (23.91-33.95)	30.18 (27.77-32.09)	**.003**	0.334	**0.023**	1.00
Waist circumference, cm	94 (85-99)	97.5 (89-113)	106 (102-115)	**<.001**	0.196	**0.003**	0.754
Metabolic syndrome, n (%)	5 (19%)	4 (15%)	32 (50%)	**<.001**	0.715	**0.005**	**0.002**
Statin use, n (%)	1 (4%)	4 (15%)	23 (35%)	**.002**	0.159	**0.002**	**0.048**
Metabolic and inflammatory characterization							
HOMA-IR	1.67 (1.05-2.51)	1.58 (1.32-2.59)	2.06 (1.49-3.43)	.086	1.00	0.271	0.726
Total-c, mg/dL	187.37 (32.31)	209.44 (29.13)	204.73 (29.13)	**.043**	0.063	0.093	1.00
LDL-c, mg/dL	118.18 (24.92)	136.81 (22.76)	129.90 (35.51)	.082	0.085	0.3	0.99
HDL-c, mg/dL	48 (41-62)	50 (45-57)	45 (39.5-52.5)	.079	1.00	0.575	0.231
his	36.68 (7.71)	39.09 (8.57)	41.09 (6.56)	**.035**	0.699	**0.033**	0.711
hs-CRP, mg/L	1.6 (0.85-2.6)	1.6 (1.15-3.75)	2.7 (1.5-6.5)	.065	1.00	0.335	0.615
Leukocytes	6590 (5700-7620)	6420 (5830-8410)	7410 (6190-8680)	.17	1.00	0.162	0.647
CV risk factors							
Dyslipidemia, n (%)	7 (26%)	9 (33%)	41 (64%)	**<.001**	0.551	**0.001**	**0.007**
Hypertension, (%)	7 (26%)	6 (22%)	932 (41%)	**.018**	0.75	**0.039**	**0.017**
Current smoker, n (%)				**.024**	0.54	**0.005**	0.14
No	14 (52%)	10 (37%)	12 (18%)				
Yes	8 (30%)	11 (41%)	30 (46%)				
Ex-smoker	5 (19%)	6 (22%)	23 (22%)				
Diabetes mellitus II, n (%)	0 (0%)	0 (0%)	7 (11%)	**.046**	-	0.076	0.076
Psoriasis characterization							
PASI	10 (8-14)	8 (5-13)	8 (5-11.5)	.059	1.00	0.156	0.380
BSA	10 (6.5-17.5)	8 (5-10)	7 (5-10)	.077	1.00	0.07	0.696
Psoriasis duration (y)	15.5 (11-31)	18 (10-30)	15 (8 -30)	.76	1.00	1.00	1.00
Psoriatic arthritis, n (%)	3 (11%)	2 (7%)	18 (28%)	**.038**	0.639	0.084	**0.032**
Atherosclerosis characterization							
Femoral atheroma plaque	0 (0%)	9 (33%)	59 (91%)	**<.001**	**0.001**	**<0.001**	**<0.001**
Carotid atheroma plaque	0 (0%)	8 (30%)	51 (78%)	**<.001**	**0.002**	**<0.001**	**<0.001**
Coronary atheroma plaque	0 (0%)	10 (37%)	51 (78%)	**<.001**	**<0.001**	**<0.001**	**<0.001**
CACS	0 (0)	3.07 (8.56)	97.03 (196.42)	**.002**	1.00	**0.013**	**0.017**
Cardiovascular scores							
SCORE2	2.1 (1.3-2.7)	2.6 (1.7-3.1)	4.5 (2.9-5.75)	**<.001**	1.00	**<0.001**	**<0.001**
QRISK3	2.2 (1.7-3.6)	3 (1.6-6.2)	8.4 (5.2-13.5)	**<.001**	0.8	**<0.001**	**<0.001**
ASCVD	1.8 (1-2.6)	3.2 (1-4.8)	7.9 (3.75-13.4)	**<.001**	0.992	**<0.001**	**<0.001**
FRS-10 y	4.51 (3.34-6.77)	6.51 (4.34-10.67)	15.13 (6.78-23.63)	**<.001**	1.00	**<0.001**	**<0.001**
PREVENT-10 y	2.65 (1.8-3.1)	3 (1.9-4.1)	7.1 (4.2-10.7)	**<.001**	1.00	**<0.001**	**<0.001**

Values reported in the table as median (IQR) for skewed quantitative variables, as mean (SD) and number (percentage) for categorical ones. Values shown in bold represent statistically significant results, defined as *P* < .005.

*ASCVD*, Atherosclerotic cardiovascular disease; *BMI*, body mass index; *BSA*, body surface area; *CV*, cardiovascular; *FRS-10 y*, Framingham 10-year risk score; *HDL*, high-density lipoprotein; *HOMA-IR*, homeostatic model of assessment for insulin resistance; *hs-CRP*, high-sensitivity C-reactive protein; *HSI*, hepatic steatosis index; *LDL*, low-density lipoprotein; *PASI*, psoriasis area and severity index; *PREVENT*, predicting 10-year risk of cardiovascular disease events; *QRISK3*, QRESEARCH, risk estimator version 3; *SCORE2*, systematic coronary risk evaluation-2.

## Results

A total of 120 patients with psoriasis were middle-aged (48.04 ± 8.25 years), and most of them were male (73.3%). The most prevalent CVRF was dyslipidemia (48.7%), followed by smoking (41.7%) and hypertension (38.3%). Patients with psoriasis had moderate-to-severe disease (median psoriasis area and severity index, 8.25 [5.5-13]), and 19.2% had psoriatic arthritis (n = 23) (Table II). One individual had noninterpretable images, and thus, 99.2% completed data for analysis.

**Table II:** Clinical, inflammatory, risk, and imaging characteristics of the Early Detection of Subclinical Atherosclerosis in Psoriasis cohort and across atherosclerosis categories (no disease, focal or 1 site affected, and multivessel disease)

	Total (N = 120)	No disease (N = 27)	Focal disease (N = 27)	Multivessel disease (N = 65)	*P* value
Clinical characteristics					
Age, y	48.04 (8.25)	41.48 (6.36)	44.40 (6.69)	52.43 (6.88)	**<.001**
Male, n (%)	88 (73.3%)	21 (78%)	18 (66.6%)	48 (74%)	.68
BMI, kg/m^2^	28.38 (25.25-32.44)	25.76 (23.60-28.27)	26.72 (23.91-33.95)	30.18 (27.77-32.09)	**.003**
Waist circumference, cm	104 (92-110)	94 (85-99)	97.5 (89-113)	106 (102-115)	**<.001**
Metabolic syndrome, n (%)	42 (35.3%)	5 (19%)	4 (15%)	32 (50%)	**<.001**
Statin use, n (%)	28 (23.3%)	1 (4%)	4 (15%)	23 (35%)	**.002**
Metabolic and inflammatory characterization					
HOMA-IR	1.86 (1.36-2.64)	1.67 (1.05-2.51)	1.58 (1.32-2.59)	2.06 (1.49-3.43)	.086
Total-c, mg/dL	202.00 (35.18)	187.37 (32.31)	209.44 (29.13)	204.73 (29.13)	**.043**
LDL-c, mg/dL	128.67 (31.10)	118.18 (24.92)	136.81 (22.76)	129.90 (35.51)	.082
HDL-c, mg/dL	47 (40-54)	48 (41-62)	50 (45-57)	45 (39.5-52.5)	.079
HSI	39.67 (7.44)	36.68 (7.71)	39.09 (8.57)	41.09 (6.56)	**.035**
hs-CRP, mg/L	2.1 (1.3-4.59)	1.6 (.85-2.6)	1.6 (1.15-3.75)	2.7 (1.5-6.5)	.065
Leukocytes	7100 (5950-8340)	6590 (5700-7620)	6420 (5830-8410)	7410 (6190-8680)	.17
CV risk factors					
Dyslipidemia, n (%)	58 (48.7%)	7 (26%)	9 (33%)	41 (64%)	**<.001**
Hypertension, (%)	46 (38.3%)	7 (26%)	6 (22%)	932 (41%)	**.018**
Current smoker, n (%)	50 (41.7%)	8 (30%)	11 (41%)	30 (46%)	**.024**
DM II, n (%)	7 (5.8%)	0 (0%)	0 (0%)	7 (11%)	**.046**
Psoriasis characterization					
PASI	8.25 (5.5-13)	10 (8-14)	8 (5-13)	8 (5-11.5)	.059
BSA	8 (6-12)	10 (6.5-17.5)	8 (5-10)	7 (5-10)	.077
Psoriasis duration (y)	16 (10-30)	15.5 (11-31)	18 (10-30)	15 (8 -30)	.76
Psoriatic arthritis, n (%)	23 (19.2%)	3 (11%)	2 (7%)	18 (28%)	**.038**
Atherosclerosis characterization					
Femoral plaque	68 (57.1%)	0 (0%)	9 (33%)	59 (91%)	**<.001**
Carotid plaque	59 (49.6%)	0 (0%)	8 (30%)	51 (78%)	**<.001**
Coronary plaque	61 (51.3%)	0 (0%)	10 (37%)	51 (78%)	**<.001**
Coronary obstructive plaque ≥ 50%	25 (21.2%)	0 (0%)	4 (15%)	21 (33%)	**.001**
CACS, n (%)	43 (35.8%)	0 (0%)	5 (19%)	37 (57%)	**<.001**
CACS (AU)	52.89 (151.33)	0 (0)	3.07 (8.52)	97.03 (196.42)	**.002**

Values reported in the table as median (IQR) for skewed quantitative variables, as mean (SD) and number (percentage) for categorical ones. Values shown in bold represent statistically significant results, defined as *P* < .005.

Comparisons between groups were conducted using analysis of variance for parametric quantitative variables or Kruskal-Wallis for nonparametric ones; comparisons regarding qualitative variables were conducted using the χ^2^ test.

*AU*, Agatston units; *BMI*, body mass index; *BSA*, body surface area; *CV*, cardiovascular; *DM*, diabetes mellitus; *HDL*, high-density lipoprotein; *HOMA-IR*, homeostatic model of assessment for insulin resistance; *hs-CRP*, high-sensitivity C-reactive protein; *HSI*, hepatic steatosis index; *LDL*, low-density lipoprotein; *PASI*, psoriasis area and severity index.

### SA in psoriasis: prevalence and relation between carotid, femoral, and coronary atherosclerosis

**Fig 1:**
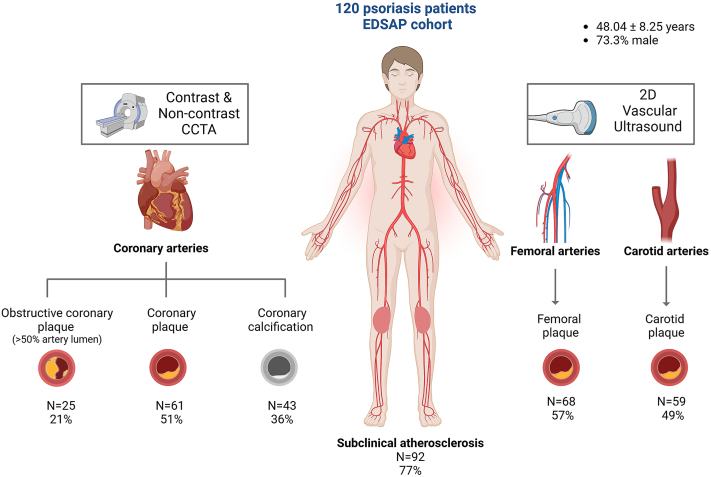
Subclinical atherosclerosis prevalence of the Early Detection of Subclinical Atherosclerosis in Psoriasis (EDSAP) cohort. Baseline imaging studies and atherosclerosis distribution for the 120 patients with psoriasis. *CCTA*, Coronary computed tomography angiography.

Overall SA prevalence was 77% (n = 92), accounting for 57.1% (n = 68) for the femorals, 51.3% (n = 61) for the coronaries, and 49.6% (n = 59) for the carotids (Fig 1). For those having coronary plaques, the mean CACS was 104.88 ± 200.65 AU, and obstructive coronary plaque was identified in 41% (n = 25). For patients with 0 CACS (n = 77), 19 (24.6%) presented nonobstructive plaques, and 7 (9%) had obstructive disease.

Further analysis of patients with obstructive coronary plaques (n = 25) showed that 76% (n = 19) had peripheral plaques, 84% (n = 21) had femoral plaques, and 76% (n = 19) had carotid plaques. There was a high NPV for excluding obstructive coronary plaques in the absence of peripheral disease (91.9%), slighter higher for the absence of femoral plaques (92.2%) compared to the absence of carotid plaques (90%). Additionally, 43.2% of patients with detectable femoral and carotid plaques were likely to have obstructive coronary plaque (Table III). In fact, the presence of femoral plaque showed the best association with all coronary parameters: obstructive coronary plaque by CCTA, as shown in Table IV.

**Table III:** Positive predictive value and negative predictive value for obstructive coronary plaque by screening femoral and carotid plaque

	Femoral and carotid plaque	95% CI
Sensitivity	76%	54.9-90.6
Specificity	73.1%	62.9-81.8
ROC area	0.75	0.65-0.84
Positive predictive value	43.2%	28.3-59
Negative predictive value	91.9%	83.2-97
	Femoral plaque	
Sensitivity	84%	63.9-95.5
Specificity	50.5%	40-61.1
ROC area	0.67	0.58-0.76
Positive predictive value	31.3%	20.6-43.8
Negative predictive value	92.2%	81.1-97.8
	Carotid plaque	
Sensitivity	76%	54.9-90.6
Specificity	58.1%	47.4-68.2
ROC area	0.67	0.57-0.77
Positive predictive value	32.8%	21-46.3
Negative predictive value	90%	79.5-96.2

*ROC*, Receiver operating characteristic.

**Table IV:** Association of femoral and carotid plaques and high-risk patients according to different cardiovascular scores with coronary atherosclerosis

	Obstructive coronary plaque		Coronary plaque presence		CACS	
	OR (95% CI)	*P* value	OR (95% CI)	*P* value	OR (95% CI)	*P* value
Femoral plaque	5.62 (1.9-20.3)	**.003**	5.02 (2.3-11.3)	**<.001**	7.95 (3.1-23.0)	**<.001**
Femoral plaque (adjusted)	2.5 (0.7-10.5)	.176	1.24 (0.4-3.3)	.682	1.96 (0.6-6.6)	.259
Carotid plaque	4.62 (1.7-13.6)	**.003**	4.56 (2.1-10.1)	**<.001**	3.27 (1.4-7.4)	**.004**
Carotid plaque (adjusted)	3.09 (1.0-10.0)	**.047**	2.65 (1.0-6.9)	**.041**	1.4 (0.5-3.7)	.506
SCORE2	1.5 (1.2-1.8)	**<.001**	1.83 (1.4-2.3)	**<.001**	1.76 (1.4-2.2)	**<.001**
PREVENT	1.2 (1.0-1.3)	**<.001**	1.49 (1.2-1.8)	**<.001**	1.35 (1.2-1.5)	**<.001**
ASCVD	1.17 (1.0-1.2)	**<.001**	1.32 (1.1-1.5)	**<.007**	1.27 (1.1-1.4)	**<.001**
FRS 10-y	1.09 (1.0-1.1)	**<.001**	1.18 (1.1-1.2)	**<.001**	1.14 (1.0-1.2)	**<.001**

Values shown in bold represent statistically significant results, defined as *P* < .005.

*ASCVD*, Atherosclerotic cardiovascular disease; *CACS*, coronary artery calcium score; *FRS-10 y*, Framingham 10-year risk score; *OR*, odds ratio; *PREVENT*, predicting 10-year risk of cardiovascular disease events; *SCORE2*, systematic coronary risk evaluation-2.

### SA by age and CVRFs

**Fig 2:**
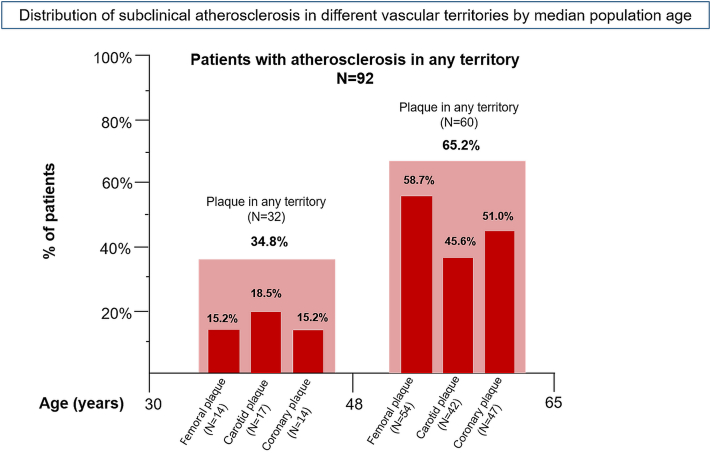
Distribution of noncoronary and coronary subclinical atherosclerosis by median population age. The distribution of patients with psoriasis with atherosclerosis (n = 92) in peripheral or coronary territories according to the median age of the Early Detection of Subclinical Atherosclerosis in Psoriasis cohort (48 years).

Multiterritorial SA distribution according to median patient age was explored, including all patients with any SA (Fig 2), being notably prevalent among younger subjects (30-48 years), accounting for 34.8% of patients with atherosclerosis with at least 1 affected territory. The prevalence and extent across all vascular territories increased with age in the 49 to 65 age group, being SA prevalence almost twice as high (65.2%).

Regarding the association between CVRFs and SA burden, the high-burden group had a significant higher median age (52.43 ± 6.88 vs 42.94 ± 6.64 years; *P* ≤ .001); higher prevalence of CVRF and metabolic syndrome (50%, n = 32 in the high-burden group vs 17%, n = 9 in the low-burden group, *P* < .001); higher insulin resistance levels (median and IQR: 2.06 [1.49-3.43] vs 1.64 [1.26-2.51]; *P* = .029); and higher high-sensitivity C-reactive protein levels (median and IQR: 2.7 [1.5-6.5] vs 1.6 [0.95-2.6]; *P* = .021) than the low-burden group (Table V). Interestingly, the femoral territory was the most frequent site with plaques in the 49 to 65 age group, whereas carotids were more commonly affected in those under the age of 48 years. Subgroup analyses were performed to examine the association of SA with psoriatic arthritis and disease duration, yielding no statistically significant results (Table VI).

**Table V:** Comparison of basal characteristics between patients with low atherosclerosis burden (no and focal disease) and patients with high atherosclerosis burden (2-5 sites affected)

	Low burden (N = 54)	High burden (N = 65)	*P* value
Demographics and clinical characteristics			
Age, y	42.94 (6.64)	52.43 (6.88)	**<.001**
Male, n (%)	39 (72%)	48 (74%)	.84
BMI, kg/m^2^	25.91 (23.91-32.14)	30.18 (27.77-32.09)	**.002**
Waist circumference, cm	95 (87-107)	106 (102-115)	**<.001**
Metabolic syndrome, n (%)	9 (17%)	32 (50%)	**<.001**
Statin use, n (%)	5 (9%)	23 (35%)	**<.001**
Metabolic and inflammatory characterization			
HOMA-IR	1.64 (1.26-2.51)	2.06 (1.49-3.43)	**.029**
Total-c, mg/dL	198.40 (32.44)	204.73 (29.13)	.33
LDL-c, mg/dL	127.5 (25.43)	129.90 (35.51)	.68
HDL-c, mg/dL	49.5 (42-57)	45 (39.5-52.5)	**.037**
HSI	37.91 (8.17)	41.09 (6.56)	**.021**
hs-CRP, mg/L	1.6 (0.95-2.6)	2.7 (1.5-6.5)	**.021**
Leukocytes	6420 (5820-7830)	7410 (6190-8680)	.063
CV risk factors			
Dyslipidemia, n (%)	16 (30%)	41 (64%)	**<.001**
Hypertension, (%)	13 (24%)	32 (49%)	**.005**
Current smoker, n (%)			**.008**
DM II, n (%)	0 (0%)	7 (11%)	**.013**
Psoriasis characterization			
PASI	9.4 (7-14)	8 (5-11.5)	**.036**
BSA	8.5 (6-15)	7 (5-10)	.09
Psoriasis duration (y)	17 (11-30.5)	15 (8 -30)	.6
Psoriatic arthritis, n (%)	5 (9%)	18 (28%)	**.011**
Atherosclerosis characterization			
Femoral plaque	9 (17%)	59 (91%)	**<.001**
Carotid plaque	8 (15%)	51 (78%)	**<.001**
Coronary plaque	10 (19%)	51 (78%)	**<.001**
Coronary obstructive plaque ≥ 50%	4 (7%)	21 (33%)	**<.001**
CACS, n (%)	5 (9%)	37 (59%)	**<.001**
CACS (AU)	1.53 (6.19)	97.03 (196.42)	**<.001**

Values reported in the table as median (IQR) for skewed quantitative variables, as mean (SD) and number (percentage) for categorical ones. Values shown in bold represent statistically significant results, defined as *P* < .005.

*AU*, Agatston units; *BMI*, body mass index; *BSA*, body surface area; *CACS*, coronary artery calcium score; *CV,* cardiovascular; *DM,* diabetes mellitus; *HDL,* high-density lipoprotein; *HOMA-IR*, homeostatic model of assessment for insulin resistance; *hs-CRP*, high-sensitivity C-reactive protein; *HSI*, hepatic steatosis index; *LDL*, low-density lipoprotein; *PASI*, Psoriasis Area and Severity Index.

**Table VI:** Atherosclerosis distribution across different clinical subtypes

	Psoriatic arthritis		*P* value	Psoriasis duration		*P* value
	No (N = 97)	Yes (N = 23)		<16 y (N = 62)	≥16 y (N = 57)	
SA presence	72 (75.0%)	20 (87.0%)	.219	47 (77.0%)	45 (78.9%)	.804
Femoral plaque	52 (54.2%)	16 (69.6%)	.18	35 (57.4%)	33 (57.9%)	.955
Carotid plaque	45 (46.9%)	14 (60.9%)	.228	27 (44.3%)	32 (56.1%)	.197
Coronary plaque	45 (46.9%)	16 (69.6%)	.051	34 (55.7%)	27 (47.4%)	.363
Obstructive coronary plaque	20 (20.8%)	9 (40.9%)	.542	15 (24.6%)	10 (17.9%)	.375

Patients were divided into groups according to the median disease duration (years).

*SA,* Subclinical atherosclerosis.

### The relationship between SA presence and CV risk scores in psoriasis

**Fig 3:**
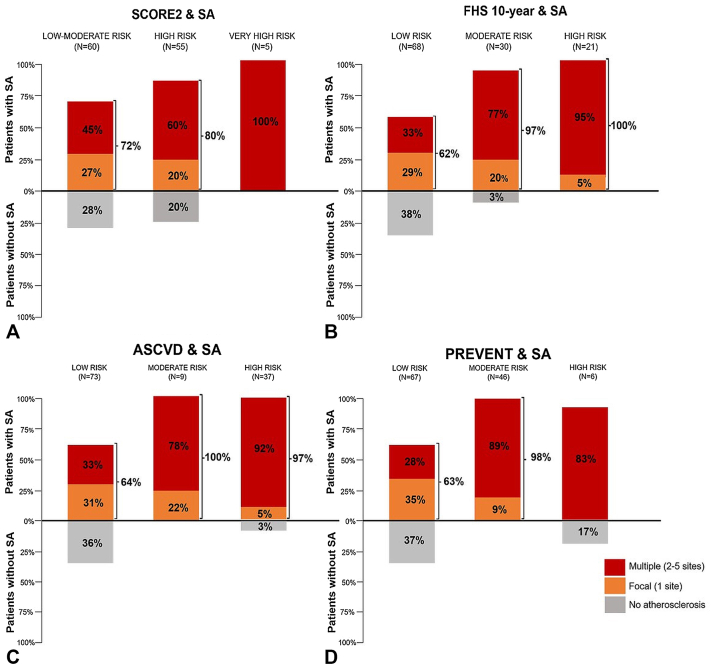
Relationship between subclinical atherosclerosis (SA) and risk categories according to clinical risk scores. **(A)** Systematic coronary risk evaluation-2 (SCORE2) risk was classified as low–moderate (<2.5%), high (≥2.5%-7.5%), or very high (≥7.5%) risk of patients <50 years; or low–moderate (<5%), high (≥5%-10%), or very high (≥10%) risk of patients >50 years. **(B)** Framingham Heart Study (FHS) 10-year risk score was classified as low (<10%), moderate (≥10%-20%), or high (≥20%). **(C)** Risk was classified as low (<5%), moderate (≥5%-7.5%), and high (≥7.5%). According to the cardiology guidelines for initiation of statin therapy, psoriasis is considered a risk-enhancing factor in patients with moderate atherosclerotic cardiovascular disease (ASCVD) risk (≥5%-7.5%). **(D)** Predicting 10-year risk of cardiovascular disease (PREVENT).

The FHS 10-year score yielded the highest risk of the whole cohort, with a 12.33%, followed by ASCVD (5.94%), PREVENT (5.78%), and SCORE2 (3.69%). As shown in Fig 3A, among patients classified as low–moderate risk by the SCORE2, 72% had SA, including 45% of high SA burden, and 80% of the high-risk patients were classified in the high SA burden group. Similarly, among participants with low FHS 10-year risk (Fig 3B), 62% had SA, and 33% of them belonged to the high-burden group. In the moderate-risk group, SA was present in 97% of the participants, with multiple territories affected in 77%. All patients in the SCORE2 very high-risk group, and the FHS 10-year high-risk group had SA. The relation between the extent of SA and the groups who met the American Heart Association/American College of Cardiology criteria for statin therapy is shown in Fig 3C. All patients classified as moderate risk (5%-7.5%) had SA, with 78% of them having multiple vascular territories affected. SA was present in 64% of the low-risk ASCVD group. Another analysis was performed to explore SA according to the PREVENT risk algorithm (Fig 3D). The results revealed a substantial prevalence of SA across all risk groups. In the low-risk group, 63% of patients exhibited SA. This prevalence increased markedly in the moderate-risk group, where 98% of participants had SA. Notably, within the moderate-risk group, nearly 89% demonstrated a high atherosclerosis burden.

## Discussion

The most important findings are as follows: (1) multiterritorial SA is highly prevalent in middle-aged patients with psoriasis; (2) femoral arteries are the most affected vascular territory; (3) the presence of femoral plaques by VUS show the strongest associations with coronary plaques by CCTA, including obstructive atherosclerosis; (4) peripheral plaque has a very high NPV for obstructive coronary plaques; and (5) a substantial proportion of patients with psoriasis, despite being classified as low risk based on CV risk scores exhibit extensive SA, underestimating the actual CV. To our knowledge, this is the most complete study evaluating SA in psoriasis, using imaging techniques with the potential to improve CV risk stratification, especially in apparently low-risk patients, providing a comprehensive assessment of SA prevalence, distribution, and burden among multiple vascular territories.

### Prevalence, vascular distribution, and burden of atherosclerosis in psoriasis: early detection as a window for preventive measures

**Fig 4:**
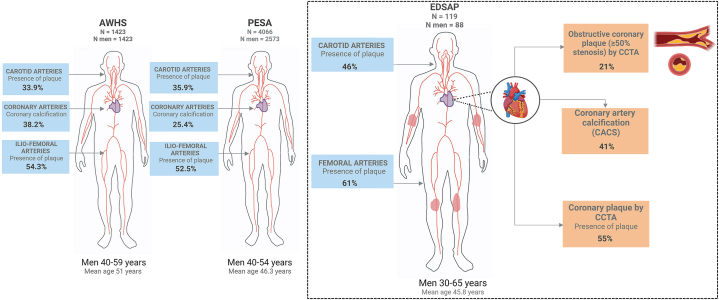
Prevalence of subclinical atherosclerosis in 3 contemporaneous cohort studies: Aragon Workers’ Health Study (AWHS), Progression of Early Subclinical Atherosclerosis (PESA), and Early Detection and Progression of Subclinical Atherosclerosis in Psoriasis (EDSAP). Prevalence of subclinical atherosclerosis by vascular territory in males in the AWHS, PESA, and EDSAP. *CACS*, Coronary artery calcium score; *CCTA*, coronary computed tomography angiography.

Multiterritorial SA prevalence in the EDSAP cohort was higher compared to that observed in general population studies, such as Progression of Early Subclinical Atherosclerosis or Aragon Workers’ Health Study (Fig 4), likely attributable to the increased CV risk of patients with psoriasis due to the systemic inflammatory nature of the disease. A high prevalence of coronary artery plaque detected by CCTA has been observed in the EDSAP cohort. Interestingly, the femoral arteries in all studies were the most frequently affected site in all these middle-aged cohorts.^5^^,^^15^ Regarding patients with a high SA burden, we found significantly higher levels of insulin resistance via the homeostatic model of assessment for insulin resistance and high-sensitivity C-reactive protein compared to patients with a low SA burden. The elevated homeostatic model of assessment for insulin resistance levels could suggest an accelerated atherogenesis partly mediated by insulin resistance mechanisms, a well-known CVRF strongly associated with psoriasis.^16^ Elevated high-sensitivity C-reactive protein, a classic biomarker of systemic inflammation, is associated with incident CV events,^17^ and has recently been proposed as a useful marker for predicting future risk of CVD in patients with psoriasis.^18^

### Femoral and carotid plaque as promising predictors for coronary atherosclerosis in psoriasis

An innovation of the EDSAP study is the early detection of coronary SA by CCTA, even in the absence of CACS. A CACS = 0 could be considered a predictor of no atherosclerosis, but among our participants, 64% had no coronary calcification, but 51% had coronary plaques, in line with Lerman et al^19^ study, showing high noncalcified coronary burden in these patients. Therefore, the absence of CACS does not necessarily indicate the absence of disease. Recently, a narrative review has stated that the use of CCTA in the context of inflammatory diseases such as psoriasis could be a better tool for CVD management in this high-risk population than other methods used before, such as CACS, because it can miss early signs of atherosclerosis before calcification happens.^1^ Beyond the detection of coronary plaque, our study leverages CCTA to additionally assess the obstructive coronary plaque, detected in 1 of 5 individuals in our sample and 41% of those with any coronary plaques.

In psoriasis, it has been stated that femoral plaque screening with VUS improves SA detection, but its association with coronary SA has not been explored.^16^ Our findings revealed a high NPV of peripheral plaque by VUS to predict obstructive coronary disease, meaning that 91.9% of individuals free of carotid or femoral plaques had no obstructive coronary plaques. The positive predictive value demonstrated that approximately 43% of patients with both femoral and carotid plaques were likely to have concomitant coronary obstruction. These results suggest that VUS of peripheral arteries may serve as a useful noninvasive screening tool to identify candidate patients for a coronary CCTA.

In our study, the femoral plaque detected by VUS showed the strongest associations with all coronary parameters by CCTA. These results align with previous findings from our group, which proposed the femoral territory as a superior predictor of SA in psoriasis.^16^ It is noteworthy to mention that, despite the high association of femoral plaque to coronary atherosclerosis, the presence of atheroma plaques in the carotids also strongly associates with coronary SA. Although the implementation of CCTA in routine clinical practice can be challenging due to accessibility, the aforementioned association between peripheral and coronary atherosclerosis suggests and reinforces that VUS screening could serve as a valuable and effective tool for the early detection of coronary SA.

### Limited value of clinical risk scores in low- to moderate-risk patients with psoriasis

CV risk in psoriasis remains significantly underestimated by conventional scores. FHS 10-year and SCORE2 scores underscore CV risk both in the general population^5^^,^^15^ and in psoriasis,^6^ which translates to a substantial excess burden of CVD and mortality. When looking at the different risk categories, all individuals at high risk had atherosclerosis. Similarly, most patients classified as moderate risk had at least 1 atherosclerotic plaque. This agrees with the statement from the 2018 American Cardiology guidelines, considering psoriasis as a risk-enhancing factor for the initiation of statin therapy in individuals classified as moderate by ASCVD.^7^ Moreover, 64% patients classified as low risk had atherosclerosis, with half of them having multiple vascular territories affected (33%). The PREVENT algorithm, developed and validated by the American Heart Association, stands out for including renal function. Nevertheless, the PREVENT risk equations continue to underestimate CVD risk in patients with psoriasis. Similar to other scores, 98% of patients classified as moderate risk had SA, with 89% of them having a high SA burden. Together, our findings provide clear evidence of SA under diagnosis in psoriasis by risk scores used in clinical practice, highlighting the need for more robust risk stratification measures in this population to better guide statin use and other preventive interventions.

The main limitations are the small sample size, the lack of a control group, and its cross-sectional nature, which has a limited capacity to evaluate clinical events. The absence of a control group or another high-risk reference group limits the possibility of defining appropriate reference values, limiting the interpretation of the magnitude and specificity of the vascular alterations observed in patients with psoriasis. In addition, patients were not stratified by clinical subtype, disease duration, or treatment status—factors that could influence the risk of SA and introduce heterogeneity. Follow-up studies will help clarify the clinical significance of early detection of SA in psoriasis. Strengths of this study are the collection of clinical and imaging data following rigorous protocols, ensuring the reliability and accuracy.

## Conclusion

Multiterritorial SA by imaging techniques was highly prevalent (77%) in this middle-aged, asymptomatic cohort of patients with psoriasis, being the femoral territory the most affected. Femoral plaques showed the strongest associations with obstructive coronary plaques as well as a high NPV, highlighting the value of screening this territory. An elevated number of patients exhibited coronary plaque despite having zero CACS, suggesting the incremental role of CCTA. Because a substantial proportion of low-to-moderate-risk participants had extensive SA, imaging of early atherosclerosis may be of interest.

## Conflicts of interest

Dr Mehta is a full-time US government employee and has served as a consultant for several pharmaceutical companies, receiving grants and/or research funding; as a principal investigator for the NIH, receiving grants and/or research funding. Dr Gelfand served as a consultant AbbVie, Artax (DSMB), BMS, Boehringer Ingelheim, Celldex (DSMB), FIDE (which is sponsored by multiple pharmaceutical companies) GSK, Inmagene (DSMB), Twill, Lilly (DMC), Leo, Moonlake (DSMB), Janssen Biologics, Novartis Corp, UCB (DSMB), Neuroderm (DSMB), and Veolia North America receiving honoraria; receives research grants (to the trustees of the University of Pennsylvania) from Amgen, BMS, and Pfizer Inc; and received payment for continuing medical education work related to psoriasis that was supported indirectly pharmaceutical sponsors. Dr Gelfand is a co-patent holder of resiquimod for the treatment of cutaneous T-cell lymphoma. Dr Gelfand is a deputy editor for the *Journal of Investigative Dermatology*, receiving honoraria from the Society for Investigative Dermatology, is the chief medical editor for Healio Dermatology (receiving honoraria), and is a member of the board of directors for the International Psoriasis Council and the Medical Dermatology Society, receiving no honoraria. Dr González-Cantero has served as a consultant for AbbVie, Janssen, Novartis, Lilly, Almirall, UCB, BMS, Celgene, and LEO Pharma, receiving grants/other payments outside the submitted work. Dr Ballester-Martínez has served as a consultant for several pharmaceutical companies, receiving grants/other payments. Dr Llamas-Velasco has served as a consultant for AbbVie, Janssen, Novartis, Lilly, Almirall, UCB, BMS, Celgene, and LEO Pharma, receiving grants/other payments outside the submitted work. Dr Berna-Rico has received honoraria from UCN and LEO Pharma outside the submitted work. Drs Abbad-Jaime de Aragon, Lecumberri, Perez-Bootello, Neria-Serrano, Monge, Pindado-Ortega, Castellanos-González, Wasniewski, Barderas, Solís, Fernández-Friera, and Jaén and Author Davó-Mogica have no interests to disclose.
